# Partners with Bad Temper: Reject or Cure? A Study of Chronic Pain and Aggression in Horses

**DOI:** 10.1371/journal.pone.0012434

**Published:** 2010-08-26

**Authors:** Carole Fureix, Hervé Menguy, Martine Hausberger

**Affiliations:** 1 Université Rennes 1, UMR CNRS 6552, laboratoire Ethologie Animale et Humaine, Rennes, France; 2 Chiropractic practice, St Jacques de la Lande, France; Centre de Recherches su la Cognition Animale - Centre National de la Recherche Scientifique and Université Paul Sabatier, France

## Abstract

**Background:**

Experiencing acute pain can affect the social behaviour of both humans and animals and can increase the risk that they exhibit aggressive or violent behaviour. However, studies have focused mainly on the impact of acute rather than chronic painful experiences. As recent results suggest that chronic pain or chronic discomfort could increase aggressiveness in humans and other mammals, we tested here the hypothesis that, in horses, aggression towards humans (a common source of accidents for professionals) could be linked to regularly reported vertebral problems of riding horses.

**Methodology/Principal Findings:**

Vertebral examination and standardized behavioural tests were made independently on the same horses. Here we showed that most horses severely affected by vertebral problems were prone to react aggressively towards humans (33/43 horses, chi-square test, df = 1, χ^2^ = 12.30, p<0.001), which was not the case for unaffected or slightly affected horses (9/16 horses, chi-square test, df = 1, χ^2^ = 0.25, p>0.05). The more affected they were, the fewer positive reactions they exhibited (rs = −0.31, p = 0.02).

**Conclusions/Significance:**

This is to our knowledge the first experimental evidence of such a link between chronic discomfort/potential pain (inferred from the presence of vertebral problems) and aggression, suggesting that chronic painful experiences may act in ways similar to those of acute experiences. Chronic discomfort or pain may often be overlooked when facing “bad tempered” individuals, whether humans or animals. This experimental study confirms the importance of including chronic discomfort or pain as a major factor in interpersonal relations and models of aggression.

## Introduction

In their unified framework/model on aggression in humans, Anderson and Bushman [1] show how personal factors (*e.g.* personality traits) and situational factors (including non-social aversive conditions, *e.g.* loud noise) can increase aggression. Amongst these factors, physical pain is one of the most powerful [2]. Aggression can increase in situations ranging from mere discomfort [3] to acute pain [4]. For instance, Anderson et al. [2] reported that moderate pain, experimentally-induced by asking subjects to hold their arm in an uncomfortable position for several minutes increased aggressive thoughts for these subjects, especially in trait hostile people (*i.e.* in whom hostility is a consistent aspect of their personality). Even in early infancy, unexpected pain may induce facial expressions [5] of anger leading Anderson and Bushman [1] to propose the existence of a “preparedness” to react aggressively when faced with physical or psychological pain, suggesting that the pain – aggression linkage could be one that humans are evolutionary prepared to develop.

Animals seem to show an increase of aggressiveness in response to acute pain as reported regularly in practice by veterinarians [6], [7]. Observations of animals' postoperative reactions to humans reveal a progressive shift from positive or neutral reactions to caregivers in the absence of pain (*e.g.* in animal anesthetized for a non-painful procedure and / or receiving analgesics) towards withdrawal and aggressive reactions (flight or fighting [8]) at higher levels of pain (*e.g.* surgical procedures without postoperative analgesic), both in dogs [9] and horses [10].

Nevertheless, these studies have focused mainly on the impact of acute rather than chronic painful experiences. Studies on the mediating processes between factors and aggression [2] and the effects of chronic pain both in humans and animals are still scarce. It has been shown that patients with chronic non-malignant pain are prone to exhibit aggressive or violent behaviour [11]. Statistics kept by the federal government indicate that 22,400 non-fatal workplace assaults were reported in the United States in 1992. Patients receiving healthcare committed 45% of these non-fatal workplace assaults and groups such as health care workers are at greatly increased risk of workplace nonfatal assaults [12]. More than 70% of patients with chronic pain expressed feelings of anger when questioned [12]. Chronic musculoskeletal disorders, especially back pain, are commonly reported in humans *e.g.* [13] and Dionne et al. [14] identified irritability and bad temper as a good predictor (among others) of failure to return to work in a study conducted to develop and to validate clinical rules to predict the 2-year work disability status of people suffering from back pain. Such a result clearly underlines a relation between chronic back pain and irritability in humans, which, of course, does not imply necessarily causal effects.

Altered welfare, as a result of chronic discomfort, is also associated with increased intra-specific aggressiveness in animals under unfavourable conditions (high social density, impoverished environment (*e.g.* in horses [15]; in pigs [16], [17]). Here we hypothesised that chronic discomfort / potential pain (inferred from the presence of vertebral problems) in riding horses is associated with increased aggressiveness towards humans. Horses share with humans a high prevalence of back problems, considered as one of the most common and least understood clinical problems in sporting horses (according to Lupton cited in [18]). Thirty-five percent of 805 race horses were found to have back problems [19], while 100% of the western horses examined by Fonseca et al. [20] suffered from thoracolumbar problems. Vertebral problems may elicit at least discomfort, and are assumed to indicate the presence of back pain [21], [22], [23]. Most of the horses affected by vertebral problems keep on working, as discomfort / potential pain is generally underestimated through lack of direct measurements and owners' personal interpretations of behaviour they assume to reflect discomfort or pain [6]. Horses are behaviourally “mute”, even when in great pain, maybe because non disclosure of pain is a valuable survival strategy to avoid predation [24], [25]. Severe back problems could be discovered after owners had observed increased aggressiveness when grooming or saddling [19] and when the horses were not just categorized as “horses with bad temper” [24]. On the other hand and independently of data for chronic painful experiences, behavioural problems with horses and especially aggression towards humans are regularly reported and constitute a common source of accidents involving veterinarians or caretakers [26]. Recent studies show that horses tend to adopt generalized attitudes towards known and unknown humans in different situations, revealing for some of them a form of “hostility state” [27].

In the present study, we investigated whether there was an association between vertebral problems, assessed by chiropractic examination, and behavioural problems, *i.e.* aggressiveness towards humans, assessed by using standardized behavioural tests in a population of 59 riding school horses of varied ages and sexes, living under similar conditions. Evaluations were made separately, each involving a different experimenter blind to the results of the other evaluation.

## Materials and Methods

Experiments complied with the current French laws (Centre National de la Recherche Scientifique) related to animal experimentation. Only behavioural observations and non painful examination were performed, as the chiropractic procedure is based on non painful handling (in the hands of a skilled manipulator) *e.g* [28], which was confirmed by the absence of any retreat behaviour of the horses. Animal husbandry and care were under management of the riding schools staffs, as this experiment involved horses from the field (no laboratory animals).

### Subjects

The 59 tested horses (44 geldings, 15 mares; 5–20 years old; mostly French saddlebred) were distributed across 3 riding centres with similar activities and housing conditions. Horses were kept singly in straw-bedded individual boxes cleaned once a day, fed industrial pellets 3 times a day and hay once a day and worked in riding lessons involving children and teenagers for 4–12 hours per week (with at least 1 rest day).

### Horses' back examination

Although all authors agree that horse back problems are very frequent, most agree also that their evaluation is difficult [19], [29], [30]. Radiographic imaging is limited by the thickness of the surrounding soft tissues [31]; ultrasonic, scientigraphic approaches all have their uses but remain difficult to apply in field conditions and on a large sample of horses [31], [32] Studying kinematics of the spine requires fixed markers and horses in controlled conditions moving in front of fixed cameras *e.g.* [33], [34], [35]. It was therefore not applicable here. Chiropractic approach clearly addresses subclinical conditions (of special interest here) and licensed professionals have an expertise in the evaluation of joints and spinal related disorders [24], [36]. Therefore, evaluation of our studied horses' spine was performed by a 20 years experienced licensed chiropractor (H. Menguy), who was totally blind to the results of the observations performed during behavioural tests and did not know the horses beforehand. Manual palpation was performed from head to tail. Manual methods have been suggested to be efficient to detect back pain [37], [38].

**Figure 1:**
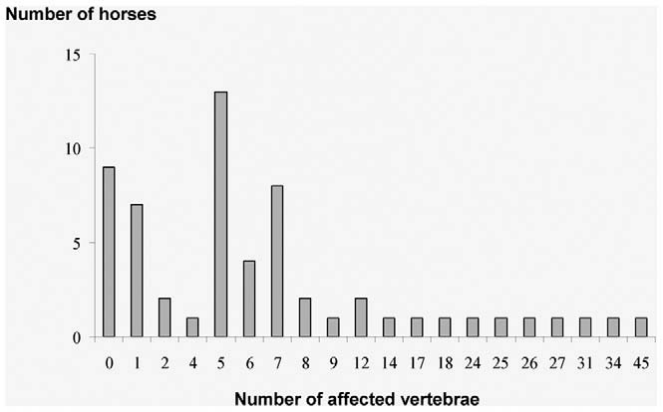
Number of horses according to their number of affected vertebrae.

Examination was based on bony and soft tissue manual palpation for localised regions of vertebral stiffness based on spinal mobilisation and palpable areas of muscle hypertonicity [28], [30]. Comparisons of data from different practitioners have shown high agreement and therefore repeatability [39]. Examinations were performed outside the horses' working times in each individual box. The horse was lightly restrained by one unknown (to the horses) experimenter (M. Hausberger) also blind to horses' results in behavioural tests. Horses were classified by the practitioner as totally unaffected, slightly affected (one slightly affected vertebra) or severely affected (at least 2 severely affected vertebrae). Very few horses had only 2 vertebrae affected (Fig. 1) and behavioural reactions confirmed this classification (see results). Data included also the number of affected vertebrae.

### Horses' reactions towards humans

Before the spine examination, horses were submitted to 5 standardized behavioural tests, routinely used in different studies on human – horse relationship. These tests were all performed by another unfamiliar experimenter (C. Fureix) outside the working times for the horses and were (see also [26], [27]):

- A motionless person test, where the experimenter entered the box and stood with her back against the closed door for 5 minutes, facing inwards and looking at the ground *e.g.* [40], [41].
- An approach contact test (based on *e.g.* [40], [42]), where the experimenter entered the box and stood motionless at 1.5m from the animal until the horse started feeding again (hay, straw), then she came closer to the animal and tried to touch its neck. She approached it from the side, walking slowly and regularly (one step per second), hands hanging by her sides, looking towards the horse's shoulder. The horse was free to withdraw. If the horse threatened the experimenter during her approach, or withdrew from her, she retreated to 1.5m from it and renewed a trial. The test was stopped when the experimenter could stroke the horse's neck continuously for 2 seconds or after three unsuccessful trials. Both sides of the horse were tested in a random order.
- A sudden approach test, where the experimenter, walking slowly along the corridor appeared suddenly at the closed door of the box while the horse was feeding (hay, straw), head down [43]. The boxes were with Dutch doors-wooden with the top and bottom divided, the bottom being solid and the top with wire gates.
- A saddle test, following the same procedure as the sudden approach test, except that the experimenter carried a saddle on her right arm and opened the box door [27].
- A halter fitting test *e.g.* [27], [44], where the experimenter entered the box, holding a halter with her left hand and approached the animal, walking slowly and regularly towards the horse's left shoulder, at approximately one step per second. When she was near the horse, she stopped walking, put her right arm over the horse's neck and fitted the halter.

Tests were performed in the same order for all horses (see [27]). Data recorded were both threats and positive reactions occurrences. Threats always consisted of ears backwards and could vary from simple threats (*i.e.* looking with ears laid back), threats to bite (*i.e.* showing the teeth in addition to simple threats) to threatening approaches (stretching the neck or approaching towards the experimenter with ears laid back); and could in some rare cases lead to real aggressions *e.g.* [43], [45], [46], [47]. No kicking or threat to kick were observed, maybe because most tests were performed with the experimenter being at the forebody level. Positive behaviours were related to investigation (looking with upright ears, approaches with upright ears, sniffing, licking, nibbling, chewing) *e.g.* [27], [40], [44]. In a previous study focused on the perception of humans by horses using the same tests described above [27], it appeared that aggressive behaviours to humans in the mere motionless person test could predict similar aggressive reactions in all other situations. Therefore, horses were classified as “aggressive” on the basis of at least one aggressive reaction in one test. Positive reaction to humans proved much less generalized and mostly associated with more invasive approaches [27]. Only some horses show consistency and therefore “positive” horses were those that showed positive behaviours (as described above) at least in 3 or more of the 5 tests.

### Statistical analyses

Analysis was conducted using Statistica^©^ 7.1 software (accepted p level at 0.05). More precise data, number of aggressive / positive reactions per test, in overall were also compared but proved mostly non significant (p>0.05 in all cases) and results will therefore not be described in details here. It seems that the presence / absence of aggressive behaviour in particular is a better indicators rather than degree. As data were not normally distributed, we used non-parametric statistical tests [48]: chi-squared tests of association, one sample chi-square tests for the relation between the horses' spine state and their reactions towards humans (as chi-squared tests of association were impossible because more than 20% of the expected frequencies are less than 5, even when pooling totally unaffected and slightly affected horses [48]) and Spearman correlation tests.

## Results

### General findings

**Figure 2:**
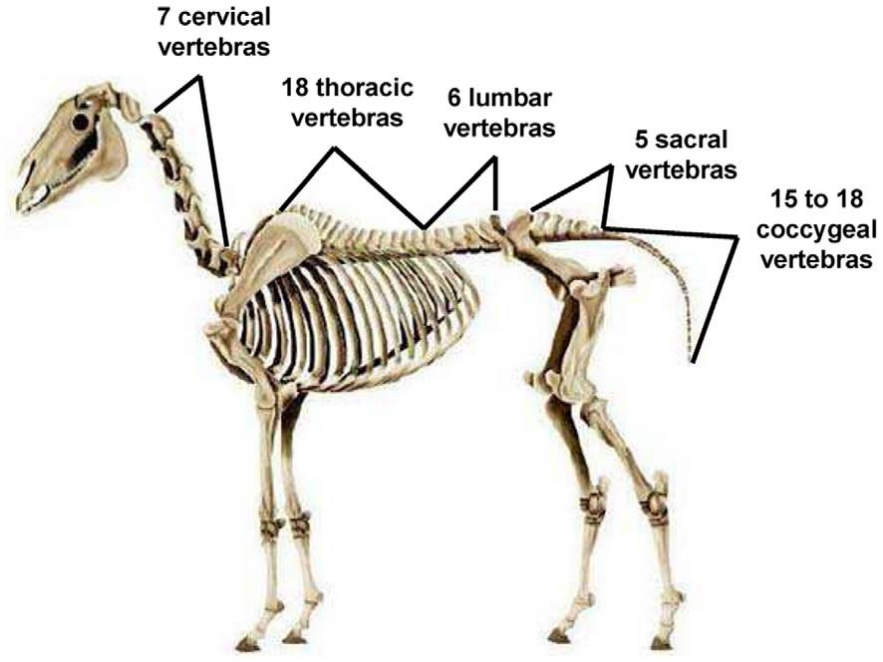
The five vertebral areas (cervical, thoracic lumbar, sacral and coccygeal) of the horse's skeleton. Adapted from Aublet [57].

**Figure 3:**
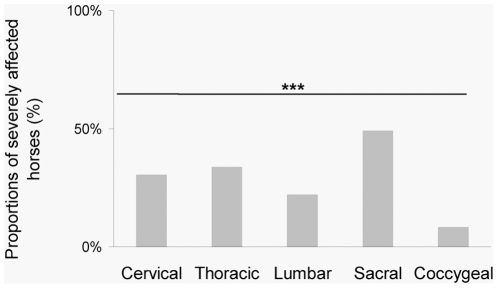
Proportions of severely affected horses in relation to vertebral area. The sacral area appeared affected in more horses than the other areas, followed by the thoracic area. Cochran test, *** p<0.001.

As in other studies *e.g.* [19], [20] most horses appeared to be severely affected (>2 severely affected vertebrae) (N = 43, 73%), while only 27% of the horses could be considered either totally unaffected (N = 9, 15%) or slightly affected (N = 7, 12%) by vertebrae problems. The sacral area appeared severely affected in more horses (49%) than the other areas (Cochran, N = 59, df = 4, Q = 30.78, p<0.001, Fig. 2 and 3), followed by the thoracic (34%), cervical (31%) and lumbar (22%) areas.

Percentage of affected vertebrae per horse varied from 0 (totally unaffected) to 88% of the whole spine (mean ± standard deviation: 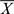 = 15.65±18.57, range: 0–88). No differences were evidenced for any of these parameters according to sex (proportions of totally unaffected, slightly and severely affected horses for males and females: chi-square, df = 2, χ^2^ = 1.15; % of affected vertebrae: n_♀_ = 15, n_♂_ = 44, 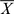
_♀_ = 19.48±18.10, 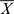
_♂_ = 14.30±18.75, U = 259.50, p>0.05 in both tests) or age (Kruskal-Wallis test, H _(2, N = 59)_ = 2,85, 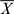
_Unaffected_ = 10±4.87, 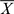
_SlAffec_ = 13.14±5.55, 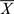
_SevAffec_ = 11.98±2.63; % of affected vertebrae: Spearman correlation test, N = 59, rs = 0.21, p>0.05 in both tests).

During the behavioural tests, 71% (N = 42) of the horses threatened the experimenter at least once (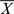 = 4.03±6.58), while 15% (N = 9) of them displayed at least one positive reaction in 3 of the 5 tests (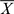 = 5.12±6.06).

### Comparison between vertebral and behavioural data

**Figure 4:**
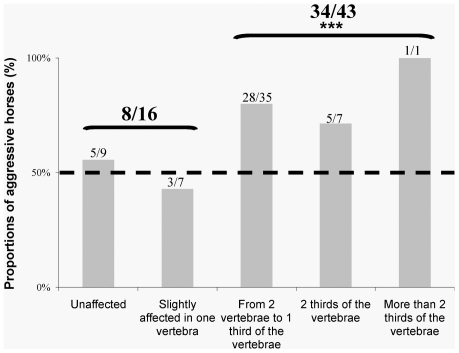
Aggressive reaction to humans in relation to the horses' spine state. Here are the proportions of horses that behaved aggressively at least once during the behavioural tests in relation to the number of affected vertebrae they had: totally unaffected (no affected vertebrae), slightly affected (only one slightly affected vertebra), from 2 severely affected vertebrae until one third of their spine (namely 17 vertebrae), 2 thirds of their spine and more than 2 thirds of the spine. Slightly affected horse reacted both similarly to totally unaffected horses and differently from severely affected horses (that were more prone to react aggressively towards humans in the tests whatever the number of affected vertebrae they have). Chi square tests, *** p<0.001.

Horses' responses in the behavioural tests clearly reflected the evaluation of their spine. Thus, while about half of the slightly affected and totally unaffected horses (8/16 horses, chi square test, df = 1, χ^2^ = 0.25, p>0.05) showed aggressiveness, more than 75% of the severely affected horses did, whether only part or most of the vertebral column was concerned (fig. 4, 34 / 43 horses, chi square test, df = 1, χ^2^ = 12.30, p<0.001). Remarkably, only 2 of the 43 severely affected horses showed consistent positive behaviour towards the experimenter (chi square test, df = 1, χ^2^ = 35.37, p<0.001), whereas about half of the other horses did (4/9 for totally unaffected horses and 3/7 for slightly affected horses, chi square test, df = 1, χ^2^ = 0.25, p>0.05).

No significant differences were observed between severely affected horses and other horses (*i.e.* unaffected or slightly affected) in the number of aggressive reactions during the behavioural tests (Mann Whitney test, U = 288, p>0.05) and the correlation between the percentage of affected vertebrae and the number of aggressive reactions was not significant (Spearman correlation test, N = 59, rs = 0.18, p>0.05).

**Figure 5:**
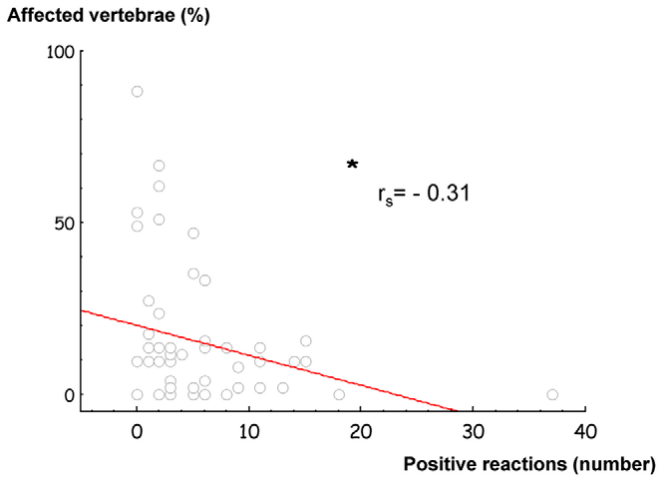
Correlation between the percentage of affected vertebrae and the number of positive reactions in behavioural tests. The more the vertebrae were affected, the less positive reactions the horses made. Spearman correlation test, * p<0.05.

However, the percentage of affected vertebrae was negatively correlated with the number of positive reactions expressed by horses towards the experimenter during the behavioural tests (Spearman correlation test, N = 59, rs = −0.31, p = 0.02, Fig. 5). Moreover, severely affected horses showed fewer positive reactions towards humans than totally unaffected or slightly affected horses (Mann Whitney test, U = 220, p = 0.03).

To sum up, relation between vertebral indicators (totally unaffected, slightly affected and severely affected horses; percentage of affected vertebrae) and horses' reactions to humans in behavioural tests converged to support a relationship between vertebral problems and aggressiveness. However, aggressive reactions were prone to appear in severely affected horses whatever to the degree of their affliction, while “positive mood” seems lowered proportionally of the degree of affliction.

## Discussion

These results, showing a clear relationship between vertebral problems, assumed to indicate the presence of chronic back pain, and aggressiveness is, to our knowledge, the first evidence of a relationship between chronic discomfort / potential pain and “bad temper” in an animal species. Finding a negative correlation between the degree of affliction and the number of positive behaviours expressed suggests a major impact of vertebral problems, not only leading them to be prone to react aggressively but also lowering considerably their “positive mood”.

Different or complementary processes may underlie this association, such as a physiological substrate common to pain and to aggression and/or association between humans and aversive events. In addition to potential morphological predispositions, vertebral disorders in horses may result from badly fitted saddles [18] or improper use of bit actions that may lead the horse to raise neck and head, inducing extension of the thoracolumbar spine [49]. Type of work *e.g* [20], [50], riding practice [39] may play a major role here. It is therefore quite possible than horses build a “memory” of these negative associations between work with humans and discomfort or potential pain, as they do for positive actions [51]. More recent data even strongly suggest an ability of horses to anticipate the positive or negative outcome of an interaction with humans [52]. Such memories are long lasting and generalized to unfamiliar humans [51], [52], [53]. Being ridden regularly while in pain may certainly lead to such associations, that evokes the “cognitive neoassociation theory” (CNT) in humans as proposed by Berkowitz et al [54]. In that theory, unpleasant experiences (here discomfort / potential pain) produce negative affect that automatically stimulates aggressive thoughts, emotional and behavioural tendencies linked together in memory. CNT thus assumes that cues present during an aversive event (here humans) become associated with the event and with the cognitive and emotional responses triggered by the event. Pain and aggression are definitely related, whether on a chronic or acute basis, but this relation may well be underestimated in cases of chronic pain. “Pain is an experience for which there is no direct measure” [6], whereas aggressive reactions are easily observed.

It seems moreover that is not as much the amount of aggressiveness as it mere occurrence that reflects vertebral problems. Therefore these problems should be listed amongst other factors known to influence personality: sire, breed, conditions of life have been shown to be involved [55]. As yet though, no study has clearly demonstrated that aggressiveness towards humans was part of a temperament profile, which seem to be more the case for positive behaviours [44]. In particular, there is no demonstration yet of a sire or breed influence *e.g.* [56]. Given the current state of knowledge, it seems more likely that aggressiveness towards humans develops in the context of daily interactions [27], [43] or as a result of the above mentioned potential lowered general state that lowers the mood in a general way.

Bearing in mind that chronic discomfort / potential pain and aggression are related may well alter the perception humans have of “bad-tempered” animals but also of other human beings. This study could increase awareness of this relationship.
